# Anti-Inflammatory, Antioxidant and Antibacterial Properties of Tomato Skin and Pomegranate Peel Extracts: A Sustainable Approach for Oral Health Care

**DOI:** 10.3390/antiox14010054

**Published:** 2025-01-05

**Authors:** Alessia Silla, Angela Punzo, Francesca Bonvicini, Matteo Perillo, Marco Malaguti, Antonello Lorenzini, Ismaela Foltran, Dario Mercatante, Mara Mandrioli, Maria Teresa Rodriguez-Estrada, Silvana Hrelia, Cristiana Caliceti

**Affiliations:** 1 Department for Life Quality Studies, Alma Mater Studiorum, University of Bologna, 47921 Rimini, Italy; alessia.silla2@unibo.it (A.S.); silvana.hrelia@unibo.it (S.H.); 2 Department of Biomedical and Neuromotor Sciences, Alma Mater Studiorum, University of Bologna, 40126 Bologna, Italy; angela.punzo2@unibo.it (A.P.); matteo.perillo2@unibo.it (M.P.); antonello.lorenzini@unibo.it (A.L.); 3 Biostructures and Biosystems National Institute (INBB), 00136 Rome, Italy; 4 Department of Pharmacy and Biotechnology, Alma Mater Studiorum, University of Bologna, 40138 Bologna, Italy; francesca.bonvicini4@unibo.it; 5 Incos-Cosmeceutica Industriale, Funo di Argelato, 40050 Bologna, Italy; ismaela.foltran@incosgroup.it; 6 Department of Agricultural and Food Sciences, Alma Mater Studiorum, University of Bologna, 40127 Bologna, Italy; dario.mercatante2@unibo.it (D.M.); mara.mandrioli@unibo.it (M.M.); maria.rodriguez@unibo.it (M.T.R.-E.); 7 Interdepartmental Centre for Renewable Sources, Environment, Sea and Energy-CIRI FRAME, University of Bologna, 40131 Bologna, Italy; 8 Interdepartmental Centre for Industrial Agrofood Research-CIRI Agrofood, University of Bologna, 47521 Cesena, Italy

**Keywords:** pomegranate peel, tomato skin, mouthwash, antioxidant activity, anti-inflammatory activity, antibacterial activity, oral care

## Abstract

Agricultural food waste and by-products could provide high-value compounds that positively affect human and environmental health, thus representing promising ingredients for cosmeceutical products. This study explores the biological activities of tomato skin (HP) and pomegranate peel (PPE) extracts on oral mucosa to evaluate their possible use in mouthwashes. The biological activities of the extracts and the mouthwash (MW) containing them were evaluated in Human Primary Gingival Epithelial cells (HGECs). The antioxidant and anti-inflammatory activities were analyzed in HGECs injured with lipopolysaccharides. After 24 h of treatment with PPE, HP, and MW, significant antioxidant activity and an increased Superoxide Dismutase 1 expression (*p* < 0.01) were observed. Additionally, the extracts significantly reduced the expression of tumor necrosis factor α (*p* < 0.05) and Monocyte Chemoattractant Protein 1 (*p* < 0.001), suggesting an anti-inflammatory role. Lastly, the antibacterial activity was assessed against *Streptococcus mutans* and *Streptococcus sanguinis* by the broth microdilution method and agar cup diffusion test for the extracts and the mouthwash, respectively, demonstrating strong effectiveness against both oral streptococcus species. Results demonstrate the potential of HP and PPE in reducing oxidative stress, inflammation, and bacterial proliferation within oral mucosa, highlighting food waste up-cycling as a resource for human health.

## 1. Introduction

To date, food sustainability is one of the most pressing global challenges facing humanity in the 21st century. With the population increase and the depletion of natural resources, adopting sustainable practices and policies is essential to ensure food security and safeguard the planet. Key measures encompass reducing food waste generation and optimizing waste management strategies.

According to the Food and Agriculture Organization (FAO), approximately one-third of all food produced for human consumption is lost or wasted annually, accounting for economic losses of 1 trillion dollars and contributing 8% of global greenhouse gas emissions (GHG) [1].

Regarding food groups, it is unsurprising that fruits and vegetables have the highest loss levels due to their perishability [2]. Moreover, not all fruit and vegetable parts are used during the processing stages, including seeds, peels, and leftover pulp, thus leading to a huge amount of residual organic waste, which can pose toxicity risks to both land and aquatic ecosystems. A more efficient management of these secondary streams could increase food sustainability and align with the United Nations Sustainable Development Goals (SDGs).

In this context, agrifood waste has recently emerged as a compelling and cost-effective reservoir of potentially functional compounds. Indeed, fruit and vegetable by-products present similar or even higher amounts of bioactive compounds, such as flavonoids, carotenoids, coumarins, etc., than edible tissues [3]. In particular, the surface tissues of fruits and vegetables are rich in carotenoids (e.g., lycopene and β-carotene), while polyphenols (e.g., flavonoids, phenolic acids, and anthocyanins) are preferentially located in peels and seeds. It is well known that these compounds are closely related to health-promoting properties [4], such as protecting cells and tissues from oxidative damage, supporting endothelial function, and preventing cardiovascular diseases [5,6].

For instance, tomato skin, usually discarded during the processing stage, has shown 2.5 times higher lycopene levels than pulp [7]. Lycopene has long been known for its several biological activities, such as reducing inflammation and redox imbalance [8].

Another example of a source of bioactive compounds is pomegranate peel, which is reported to contain a 10-fold higher phenolic content than the pulp [9]. Some polyphenols, such as punicalagin and punicalin, are characteristic of this fruit and have health-beneficial properties, mainly in inflammation-associated chronic diseases [10,11].

Driven by their beneficial properties, the demand for functional products enriched with bioactive compounds is rapidly rising. Therefore, the upcycling of agrofood waste as a source of these ingredients can significantly benefit the health industries, unlocking new attractive opportunities and promoting sustainable development.

In this context, oral hygiene products present a promising field underexplored to study the potential benefits of agrofood waste-derived compounds. Oral diseases, such as gingivitis and periodontitis, are increasingly prevalent worldwide, affecting approximately 3.5 billion individuals [12]. Despite their potential to impact systemic health [13], these conditions remain significantly underestimated and are often inadequately managed.

Indeed, oral bacteria residing in dental plaque play a key role in the pathogenesis of these diseases, and recent studies pointed out the equally significant—or potentially more critical—contributions of oxidative stress and inflammation to disease progression [14]. For instance, gingivitis, which may evolve into periodontal disease, characterized by gingival, ligament, and bone destruction, is typically initiated by specific aerobic facultative bacteria within subgingival plaque, such as *Streptococcus sanguinis*. In this regard, bacterial lipopolysaccharides (LPSs) stimulate the release of various cytokines (i.e., tumor necrosis factor (TNF)-α and Interleukin (IL)-1β), initiating an inflammatory cascade that promotes bone resorption [15,16]. Concomitantly, there is an overproduction of reactive oxygen species (ROS) that decreases collagen content and increases matrix metalloproteinase levels, promoting the degradation of connective tissue and the bone matrix [16].

Proper oral hygiene practices are crucial for oral disease prevention and treatments, highlighting the importance of effective oral home-care products, such as mouthwashes. Enhancing these formulations with biologically active compounds—possessing antioxidant, anti-inflammatory, and antibacterial properties—may reduce the risks of dental decay and counteract the onset and progression of oral diseases.

In this context, we exploited the biological activities of pomegranate peel (PPE) and tomato skin (HP) extracts to evaluate their possible use as components in mouthwashes and, in general, for oral care products.

## 2. Materials and Methods

### 2.1. Chemicals

Phosphate-buffered saline (PBS) tabs (giving a 137 mM NaCl, 2.7 mM KCl phosphate-buffer solution, pH 7.4, final concentration: 0.01 M), trypsin–EDTA, an antibiotic solution 100× (10,000 U/mL penicillin and 10 mg/mL streptomycin), gallic acid, resveratrol, Curcumin, Ampicillin sodium salt, Folin and Ciocalteu’s phenol reagent, and the oxidative stress inductor lipopolysaccharide (LPS) were obtained from Sigma-Aldrich (St Louis, MO, USA). A mother solution of LPS (5 mg/mL) was prepared in PBS and stored at −20 °C.

Human Primary Gingival Epithelial cells, a Complete Human Epithelial Cell Medium, and a gelatin-based coating solution were purchased from CliniSciences (Nanterre, France). Fetal Bovine Serum (FBS); a Trypan Blue solution, 0.4%; and a Power SYBR™ Green RNA-to-CT™ 1-Step Kit were acquired from Thermo Fischer Scientific (Waltham, MA, USA). The WST-8 kit and the cytotoxicity lactate dehydrogenase (LDH) assay kit were acquired from Dojindo Molecular Technologies (Kumamoto, Japan).

A RNeasy Mini Kit was purchased from QIAGEN (Hilden, Germany). Primers for Real-Time PCR were purchased from IDT (Coralville, IA, USA).

The chemiluminescent probe (AquaSpark™ 510 Peroxide Probe) was kindly provided by Biosynth Carbosynth (Staad, Switzerland). A mother solution of 10 mM was prepared by solubilizing the probe in DMSO. It remains stable for months when stored at 4 °C protected from light. All the other chemicals and solvents were of the highest analytical grade.

### 2.2. Extracts and Mouthwash Formulation

A Hydropom^®^ extract (HP) is a water-based lycopene-containing product produced by Phenbiox srl. (Calderara di Reno, BO, Italy). Hydropom^®^ was obtained through patented A.M.C. (Aquose Microdisperse Carotenoids) technology, combining the bioliquefaction technologies with the micro-dispersion of oil-soluble molecules in the water phase. It additionally contains citric acid, sodium benzoate, and potassium sorbate.

According to the manufacturer, the product exhibited a total sugar content of 4.0 ± 1.0 g/kg, a phenolic concentration of 0.1 ± 0.05 g/kg, and a lycopene concentration of 25.0 ± 5.0 mg/L.

The *Punica granatum* L. peel extract (PPE) was produced by Phenbiox srl. (Calderara di Reno, BO, Italy). This included peel extract (22.5% *w*/*v*), *Saccharomyces* ferment lysate filtrate, citric acid, sodium benzoate, and potassium sorbate.

Before the use, both extracts were filtered in 0.22 µm polytetrafluorethylene (PTFE) membranes and maintained at 4 °C.

The mouthwash final formulation containing both extracts at 3% was kindly supplied by Coswell (Funo, Bologna, Italy). Due to its viscosity, the mouthwash was filtered in 0.22 µm polytetrafluorethylene (PTFE) membranes and then maintained at 4 °C. The working solutions were prepared immediately before the experiments, after the appropriate dilution of the extracts within the cell culture medium, according to the instructions for use provided by manufacturers.

### 2.3. Evaluation of Total Phenolic Content

The total phenolic content (TPC) of PPE was evaluated through the Folin–Ciocalteu reagent assay previously described by Ainsworth et al. [17].

In summary, 100 μL of the extract solution was combined with 200 μL of the Folin–Ciocalteu reagent. Subsequently, 800 µL of a 700 mM sodium carbonate solution was added, and the reaction mixture was incubated for 2 h at room temperature. Following incubation, 200 µL of each sample was transferred into a 96-well microplate, and the absorbance of each well was determined spectrophotometrically at 765 nm using distilled water as a blank. The results were averaged from three measurements. Gallic acid (range: 100–1000 μg/mL) was used as a standard, and the TPC was expressed as µg of gallic acid equivalent (GAE)/mL pomegranate peel extract.

### 2.4. HPLC-UV Determination of Punicalagin

As suggested by Qu et al., a reversed phase method was utilized for the high-performance liquid chromatography (HPLC) separation and quantification of punicalagin content in PPE [18] with some modifications. The determination of punicalagin was performed by using an HPLC-UV (HPLC LC-2030C, Shimadzu, Kyoto, Japan) equipped with a C18 column (SH-SPP C18LPH, 150 mm × 4.6 mm × 2.7 µm, Shimadzu), a guard column (SH-EXP, 5 mm × 4.6 mm × 2.7 µm, Shimadzu), a UV detector, and data acquisition software (LabSolutions version 5.84, Shimadzu, Kyoto, Japan). The eluent mixture was water + 0.1% phosphoric acid (A) and acetonitrile (B). The analyses were run at a flow rate of 1.0 mL/min, under the following gradient conditions: 98% of A up to 20 min, 90% of A up to 30 min, 70% of A up to 32 min, and 98% of A up to 42 min. The column was thermostated at 35 °C, and the autosampler was thermostated at 4 °C. Before the analysis, the pomegranate peel extract was diluted with methanol, to ensure a good solubilization of punicalagin and a good peak resolution. PPE was filtered through a 0.2 μm PTFE membrane before being injected into the HPLC-UV instrument. The injection volume was 10.0 μL and UV detection was set at 378 nm.

The quantification of punicalagin was performed using calibration curves constructed with an external standard (punicalagin A + B mixture, PhytoLab GmbH & Co. KG, Vestenbergsgreuth, Germany), with solutions in a concentration range of 5–125 μg/mL. Linear regression equations and correlation coefficients were calculated for punicalagin A and B (y = 3011.2x − 5413.2, R^2^ = 0.9979 (punicalagin A); y = 3820.8x – 17,750, R^2^ = 0.9905 (punicalagin B)). The limits of detection (LODs) and quantification (LOQs) for punicalagin A and B were calculated as 3- and 6-fold the standard deviation of the intercept of the calibration curve [19]. All analyses were performed in triplicate.

### 2.5. Gingival Cell Model

Human Primary Gingival Epithelial cells (HGECs), isolated from normal human gingival tissue, were purchased from CliniSciences (Nanterre, France). Cells were grown in tissue culture flasks pre-coated with a gelatin-based coating solution and maintained in a Complete Human Epithelial Cell Medium containing 10% FBS, and growth factors, at 37 °C in an atmosphere of 5% CO_2_.

### 2.6. Cell Viability Bioassay

Cell viability was determined using a WST8 [2-(2-methoxy-4-nitrophenyl)-3-(4-nitrophenyl)-5-(2,4-disulfophenyl)-2H-tetrazolium, monosodium salt] assay. In this assay, WST8 is reduced by cellular dehydrogenases, producing a soluble formazan dye that is soluble in the culture medium. The quantity of formazan is directly proportional to the number of living cells. Absorbance was measured at 450 nm using an AMR-100 Microplate Reader (Allsheng, Hangzhou, China) comparing the treatments to the control.

For this set of experiments, HGECs were seeded in a 96-pre-coated-well plate (10 × 10^4^ cells/well) and grown at 37 °C, 5% CO_2_. After 24 h, cells were treated for 4 h with serial dilutions of PPE in the concentration range of 0.5–3% *v*/*v* (range in content of punicalagin: 7.72–46.36 µg/mL), HP in the range of 0.5–3% *v*/*v* (range in content of lycopene: 0.12–0.75 µg/mL), and a solution containing both extracts (PPE + HP) at the concentration of 3% *v*/*v* with a punicalagin content of 46.36 µg/mL and lycopene content of 0.75 µg/mL. The decrease in absorbance between 4 h treatments and control was monitored at 37 °C at 450 nm using an AMR-100 Microplate Reader.

### 2.7. Cell Cytotoxicity: Lactate Dehydrogenase Release

The detection of lactate dehydrogenase (LDH) released by HGECs was carried out using a standard spectrophotometric method through the collection of cellular medium aliquots [20]. The assay involves a coupled enzyme reaction: LDH catalyzes the conversion of lactate to pyruvate, concurrently reducing NAD+ to NADH. Simultaneously, a diaphorase enzyme reduces a tetrazolium salt to generate a red formazan product through the oxidation of NADH. The quantity of formazan, measurable at 490 nm, is directly proportional to the LDH content in the culture, providing a direct reflection of the number of dead or damaged cells.

For these experiments, the HGEC medium was obtained from HGECs treated for 4 h with different concentrations of PPE (0.5–3% *v*/*v*), HP (0.5–3% *v*/*v*), and PPE + HP (3% *v*/*v*), and collected. The increase in absorbance between the treatments and the control was monitored at 37 °C using an AMR-100 Microplate Reader.

### 2.8. Trypan Blue Exclusion Test

Given the opalescence of the mouthwash formulation, which could have affected the spectrophotometer analysis, cell viability was also evaluated with the Burker chamber using the Trypan Blue exclusion test.

For the assay, HGECs were seeded in six-pre-coated-well plates (1 × 10^6^ cells/well) and grown at 37 °C, 5% CO_2_. After 24 h, cells were incubated for 4 h with different concentrations of PPE (0.5–3% *v*/*v*), HP (0.5–3% *v*/*v*), PPE + HP (3% *v*/*v*), and a mouthwash formulation (MW) containing both extracts at the concentration of 3% *v*/*v* in the dilution of 1:2 (content of punicalagin: 23.18 µg/mL, content of lycopene: 0.37 µg/mL). The mouthwash was not used undiluted due to its slightly opaque and viscous consistency, which could interfere with the accuracy of the analysis.

Next, the cell number was manually counted using the Burker chamber after the staining with the Trypan Blue solution (0.2% in PBS).

### 2.9. Antioxidant Activity of the Extracts and Mouthwash for Mulation on HGECs

The intracellular H_2_O_2_ production was estimated in HGECs by the chemiluminescent (CL) cell-based bioassay previously described by our group [21]. The working solutions of the CL probe and the LPS were prepared by diluting the respective stock solutions with PBS.

Firstly, the correlation between the CL emission and the production of H_2_O_2_ was investigated in HGECs stimulated with different concentrations of LPS as oxidative stress inductors.

The day before the experiment, HGECs (10 × 10^4^ cells/well) were plated in a 96-well black microtiter plate with a transparent bottom. After 24 h, 50 μL of the CL probe working solution was added to each well and then incubated for 20 min at 37 °C to obtain a final 5 μM/well concentration. Then, 50 μL of serial dilutions of standard solutions of LPS (5 mg/mL) were dispensed to induce intracellular H_2_O_2_ production (final concentration range: 2.5–50 μg/mL). The PBS solution was used as a negative control. The CL emission was observed for 40 min through a Luminoskan™ Ascent luminometric plate reader (Thermo Fisher Scientific, Roskilde, Denmark) [21]. The whole assay was performed at 37 °C. The dose–response curve was achieved by plotting the CL signal versus the LPS concentration and fitting the experimental data to a straight line using the least squares method.

Once the method was optimized in HGECs, the extract and mouthwash formulation antioxidant activities were investigated.

A total of 24 h before the experiment, HGECs (10 × 10^4^ cells/well) were plated in a 96-well black microtiter plate with a transparent bottom.

After 24 h, cells were treated for 4 h with serial dilutions of PPE (0.1–3% *v*/*v*), HP (0.1–3% *v*/*v*), PPE + HP (0.1–3% *v*/*v*), MW at the dilution range of 1:2–1:4, and resveratrol (R) (concentration range: 0.1–25 μM) as positive antioxidant control [22]. The PBS solution was used as a negative control. Then, 50 μL of the CL probe working solution (final concentration: 5 μM/well) was added to each well and incubated for 20 min at 37 °C. After the incubation, 50 µL of LPS at a fixed concentration (25 µg/mL) was added to each well. The CL emission signal was monitored using a Luminoskan™ Ascent luminometric plate reader. The whole assay was performed at 37 °C. The dose–response curve was obtained by plotting the CL signal versus the extract treatment concentration.

### 2.10. RNA Extraction

Two days before the experiment, HGECs (1 × 10^6^ cells/well) were seeded in six-pre-coated-well plates and maintained in a Complete Human Epithelial Cell Medium at 37 °C in an atmosphere of 5% CO_2_. The following day, HGECs were pretreated for 4 h with serial dilutions of PPE (0.5–3% *v*/*v*), HP (0.5–3% *v*/*v*), PPE + HP (3% *v*/*v*), MW at the dilution 1:2 and 1:3, R (10 μM), and Curcumin (C) (concentration of 10 μM) as positive anti-inflammatory control [22,23]. Next, the culture medium was replaced, and cells were injured with LPS (25 μg/mL) for 16 h.

Total RNA extraction was performed using the commercial kit RNeasy Mini Kit according to the manufacturer’s recommendations. Then, RNA concentration and purity were determined spectrophotometrically using the Nanodrop 1000 spectrophotometer (Thermo Fisher Scientific, Waltham, MA, USA).

### 2.11. Real-Time PCR

Total RNA (25 ng) was reverse-transcribed using the Power SYBR™ Green RNA-to-CT™ 1-Step Kit according to the manufacturer’s recommendation in a final volume of 20 μL.

Reaction conditions were conducted on a QuantStudio1 Real-Time PCR System (Thermo Fisher Scientific, Waltham, MA, USA), with an initial reverse transcription of 10 min at 45 °C, then 2 min at 95 °C, followed by 40 cycles of amplification (95 °C for 5 s and 60 °C for 20 s), and evaluated by QuantStudio 1 Real-Time PCR System Software. Primer concentration was 50 μM. The following human primers were used:

RPL13A forward 5′-CACCCTGGAGGAGAAGAGGA-3′, reverse 5′-CCGTAGCCTCATGAGCTGTT-3′.

SOD-1 forward 5′-AGGCATGTTGGAGACTTGGG-3′, reverse 5′-TGCTTTTTCATGGACCACCAG-3′.

TNF-α forward 5′-CCATGTTGTAGCAAACCC-3′, reverse 5′-GAGTAGATGAGGTACAGGC-3′.

MCP-1 forward 5′-GATCTCAGTGCAGAGGCTCG-3′, reverse 5′-GGTCTTGAAGATCACAGCTTCTT-3′.

Changes in gene expression levels were analyzed by the 2^−ΔΔCt^ formula [24], and the RPL13A (ribosomal protein L13-A) was used as the reference gene.

### 2.12. Antibacterial Activity

#### 2.12.1. Broth Microdilution Assay

The antibacterial activity of HP and PPE was evaluated against *Streptococcus mutans* (ATCC 35668) and *Streptococcus sanguinis* (ATCC 10556) using a well-established broth microdilution procedure in 96-well microtiter plates and compliance with the Clinical and Laboratory Standard Institute (CLSI) guidelines. Briefly, bacterial inocula were prepared at 0.5 McFarland in PBS and, subsequently, suspensions were diluted at 1:200 in Brain Heart Infusion Broth (Biolife Italiana S.r.l, Monza, Italy) and incubated with serial dilutions of extracts in the range 0.3125–10% *v*/*v*. Positive controls (bacterial suspension in regular medium), negative controls (only medium), and background controls (extracts’ dilutions in regular medium) were included in the tests. The commercially available ampicillin (Ampicillin sodium salt) was used as a reference drug at the inhibitory concentration of 0.5 µg/mL, as reported in the EUCAST Clinical Breakpoint Tables (v. 14.0, valid from 1 January 2024).

The plates were incubated at 37 °C for 24 h, and subsequently, the optical density at 630 nm (OD_630nm_) was spectrophotometrically measured. Next, OD_630nm_ values were calculated for each tested dilution, and data were expressed as percentage values relative to the positive controls. The MIC (Minimum Inhibitory Concentration) was defined as the lowest dilution (% *v*/*v*) at which bacterial growth was <10%.

Each experiment was carried out with three technical replicates (i.e., three wells per sample) and repeated at least two times for statistical power.

#### 2.12.2. Agar Well Diffusion Method

The antibacterial activity of MW was assayed in vitro against *S. mutans* and *S. sanguinis* by using an agar cup diffusion method carried out on Mueller–Hinton agar (MHA) plates (Biolife Italiana S.r.l, Monza, Italy). The bacterial inocula were prepared as previously described and spread on the MHA plates. A cork-borer was used for making wells on the agar plate (6 mm in diameter), and each well was subsequently filled with 100 µL of a test sample. The plates were incubated for 24 h at 37 °C and the diameter of the inhibition zone was measured, with a ruler, to the nearest whole millimeter. The commercially available ampicillin (Ampicillin sodium salt) was used as a reference drug at the inhibitory concentration of 0.5 µg/mL, as reported in the EUCAST Clinical Breakpoint Tables (v. 14.0, valid from 1 January 2024). All experiments were performed in duplicate on different days.

## 3. Results and Discussion

### 3.1. Phenolic Compound Profile of Pomegranate Peel Extract

Pomegranate peel extract is usually renowned for its high content of phenolic compounds, including flavonoids and hydrolyzable tannins (mainly ellagitannins such as punicalagin), each being well known for their benefits on human health. The Folin–Ciocalteu assay was performed in PPE, showing a high polyphenol concentration (1888.89 ± 8.14 µg GAE/mL). These data are in line with the literature, confirming the presence of abundant levels of polyphenols in pomegranate peel [25].

### 3.2. Quantitative Analysis of Punicalagin in the Pomegranate Peel Extract

**Figure 1:**
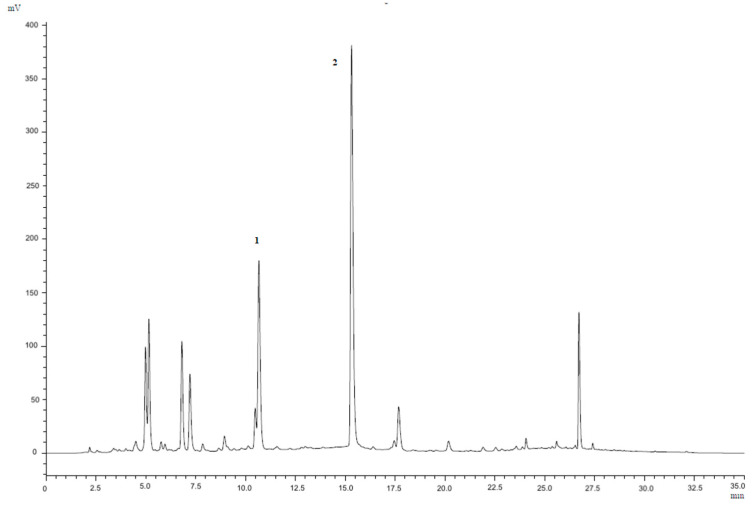
Reversed phase HPLC-UV chromatogram of PPE at 378 nm. Peak identification: 1, punicalagin A; 2, punicalagin B.

Firstly, the quantification of punicalagin, the major polyphenol present in pomegranate peel [26], was carried out by HPLC-UV. Figure 1 shows the HPLC-UV trace of the PPE at 378 nm, where punicalagin A and B were detected and quantified. The content of punicalagin in the PPE extract was 1545.45 ± 12.23 μg/mL, of which 692.93 ± 29.07 μg/mL was punicalagin A and 852.42 ± 40.55 μg/mL was punicalagin B. The LOD and LOQ of punicalagin A were 4.79 μg/mL and 8.70 μg/mL, whereas the LOD and LOQ of punicalagin B were 10.30 μg/mL and 18.73 μg/mL.

### 3.3. The Safety Evaluation of the Extracts in the Human Gingival Epithelium Model

The safety and biological activities of the extracts were evaluated on primary epithelial cells isolated from human gingival tissue (HGEC). Indeed, they provide highly predictive human models, thus minimizing animal use in scientific research. The gingival epithelium acts as a first barrier, playing a key role in the oral defense against different insults, from microbial challenges to oxidative damage. Given these features, HGECs provide valuable in vitro models to ensure the safety and efficacy of oral products.

**Figure 2:**
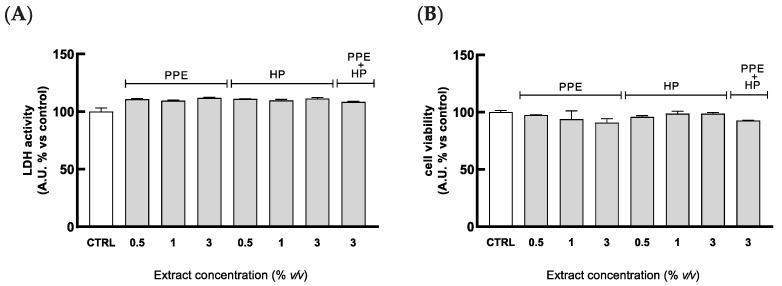
Effects of PPE and HP extracts on cytotoxicity and cell viability. HGECs were pretreated for 4 h with serial dilutions of PPE (range in content of 7.72–46.36 µg/mL), HP (range in content of lycopene: 0.12–0.75 µg/mL), and PPE + HP (content of punicalagin: 46.36 µg/mL, content of lycopene: 0.75 µg/mL). (**A**) Cytotoxicity was quantified by spectrophotometrically measuring LDH released in the cell medium. (**B**) Cell viability was assessed by measuring red dye production at 490 nm. Ctrl (control, untreated cells). Results are expressed as the mean ± SD of three independent experiments.

Firstly, HGECs were pretreated for 4 h with serial dilutions of PPE (0.5–3% *v*/*v*), HP (0.5–3% *v*/*v*), and PPE + HP (3% *v*/*v*), to evaluate their potential cytotoxicity. As shown in Figure 2A, all the extract dilutions (alone or combined) were not cytotoxic toward the cells, as indicated by the absence of significant alterations in LDH release in treated cells compared to the control. Higher concentrations, up to 5%, were cytotoxic when the formulations were combined. Moreover, the same treatments do not reduce cell metabolism, showing any changes in the formazan dye levels between samples and control, as illustrated in Figure 2B.

Next, the safety of the investigated solutions was assessed by counting viable cells using Trypan Blue dye as the exclusion test. Indeed, an additional assay was required to assess the safety of the mouthwash formulation, which is an opalescent solution containing particles, thus affecting measurements with the spectrophotometer reader.

**Figure 3:**
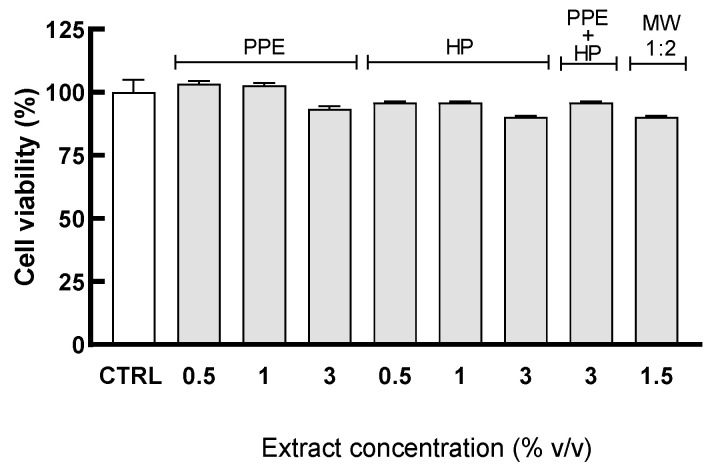
The Trypan Blue exclusion test in HGECs treated with PPE and HP extracts and the mouthwash. HGECs were pretreated for 4 h with serial dilutions of PPE (range in content of 7.72–46.36 µg/mL), HP (range in content of lycopene: 0.12–0.75 µg/mL), PPE + HP (content of punicalagin: 46.36 µg/mL, content of lycopene: 0.75 µg/mL), and MW at the dilution 1:2 (content of punicalagin of 23.18 µg/mL, content of lycopene of 0.37 µg/mL). Cell viability was assessed by counting viable cells using Trypan Blue dye as the exclusion test. Results are expressed as the mean ± SD of three independent experiments.

HGECs were pretreated for 4 h with the extracts PPE (0.5–3% *v*/*v*), HP (0.5–3% *v*/*v*), and PPE + HP (0.5–3% *v*/*v*) and MW at the dilution 1:2. As shown in Figure 3, none of the treatments significantly reduce cell viability. These results are in line with data obtained from the previous bioassays, showing good biocompatibility with the HGEC model.

Moreover, since the extracts are commercially available and cosmetic-grade ingredients, they comply with the relevant safety and quality standards for cosmetic applications as outlined in the European Union’s Regulation (EC) No. 1223/2009. This ensures that the formulations are both effective and safe for their use in oral health formulations, aligning with current regulatory requirements and promoting sustainable, eco-friendly practices.

Therefore, we conducted an additional analysis on both extracts and the mouthwash formulation to investigate their antioxidant, anti-inflammatory, and antibacterial effects on HGECs.

### 3.4. Evaluation of Antioxidant Effects of Extracts in Human Gingival Epithelial Cells

Oxidative stress reflects a cellular imbalance caused by ROS excess and insufficient detoxifying systems. In physiological conditions, ROS generated by gingival cells are rapidly removed by non-enzymatic and enzymatic antioxidant complexes, thus protecting the oral mucosa.

The presence of an infection or disease in the oral cavity, tobacco use, and bad eating habits can promote a ROS overproduction. High levels of these free radicals result in a cascade of events, such as cartilage proteoglycan and glycosaminoglycan degradation, mediating progressive periodontal tissue destruction [27].

Using a recently developed chemiluminescent and quantitative bioassay selective for quantifying intracellular H_2_O_2_ production [21], we first monitored the H_2_O_2_ levels in HGECs upon treatment with the pro-oxidant agent LPS. LPS is the major component of the Gram-negative bacteria cell wall, generally considered a pro-inflammatory stimulus able to trigger ROS production in gingival tissues [28].

**Figure 4:**
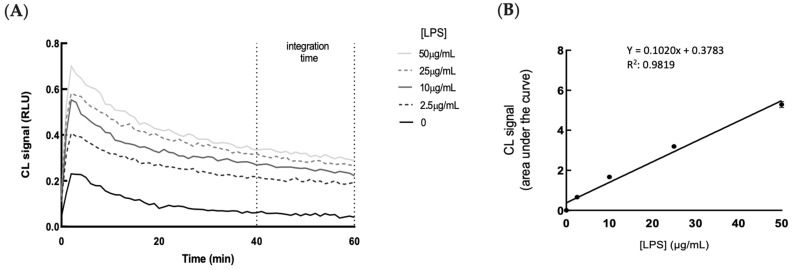
Kinetic profiles of H_2_O_2_ production in HGECs treated with LPS by using a chemiluminescent method. (**A**) CL kinetic profiles were acquired for HGEC cells upon CL probe incubation and treatment with different concentrations of LPS (2.5–50 µg/mL). (**B**) The dose–response curve showing the correlation between the CL signal and LPS concentration. Each point represents the mean ± SD of three independent measurements.

Figure 4A,B show the kinetic profiles of the CL emission and the correlation between the CL signal and LPS concentration in HGECs, respectively. As expected, the CL emission intensity linearly increased in the presence of increased concentrations of LPS. 

Next, HGECs were pretreated for 4 h with PPE (0.1–3% *v*/*v*), HP (0.1–3% *v*/*v*), PPE + HP (0.1–3% *v*/*v*), MW (dilution range of 1:2–1:4), and R (0.1–25 μM) as positive antioxidant control. Then, cells were injured with LPS (25 μg/mL), and the CL signal was monitored.

As shown in Table 1, all tested solutions significantly reduced intracellular H_2_O_2_ production, obtaining IC_50_ values of 0.36 ± 0.01% *v*/*v* for PPE, 0.34 ± 0.02% *v*/*v* for HP, 0.23 ± 0.02% *v*/*v* for PPE + HP, and 0.25 ± 0.02% *v*/*v* for MW.

According to previous studies on other cellular models [29], these results suggest a good antioxidant activity of pomegranate and tomato by-products have good antioxidant activity, possibly linked to their high polyphenol content. Interestingly, combining extracts into the same solution or formulation enhances antioxidant efficacy. This may be explained by the different bioactive molecule profiles of the extracts. For instance, pomegranate peel has a high ellagitannin content (mainly punicalagin and punicalagin derivatives), while tomato skin is particularly rich in carotenoids such as lycopene. The different chemical compositions result in different pathway activations to counteract free radical overproduction [30,31]. The possible synergistic/additive action of these compounds, as well as the multiple-antioxidant-path involvement, may improve the outcomes, showing a lower IC_50_.

Resveratrol, known for its potent antioxidant properties [22], showed an IC_50_ of 0.05 µg/mL, which is considerably lower than all tested extracts, confirming its efficacy. However, the combined treatment of PPE and HP approaches this level of effectiveness, suggesting that the possible synergistic interaction between punicalagin and lycopene may significantly enhance antioxidant capacity compared to the activity of each single extract.

Consequently, the integration of both PPE and HP (3% *v*/*v*) into the MW formulation, which showed a low IC_50_ too, reinforces its potential as a promising option for both therapeutic and cosmetic purposes by effectively leveraging the synergistic effects of the extracts.

Additionally, supplementary experiments demonstrated that saliva slightly increased the IC_50_ values of PPE and HP. However, the IC_50_ values remained low at higher or combined concentrations. These results indicate that, although saliva moderates the activity, the extracts retain significant antioxidant potential, supporting their cosmetic applicability under physiologically relevant conditions (see Supplementary Materials, Table S1).

### 3.5. Effects of Extracts on Superoxide Dismutase 1 Levels in HGEPs Injured with LPS

Given the role of oxidative stress in periodontal pathologies [32], improving antioxidant defense and maintaining cell homeostasis are crucial for oral health. Superoxide dismutase (SOD) is one of the most important families of antioxidant systems for scavenging free radicals and protecting the tissues (including oral mucosa) against ROS damage. SODs catalyze the dismutation of O_2_^•−^ into oxygen and H_2_O and play a key role in inhibiting the oxidative modification of nitric oxide, thus preventing peroxynitrite (ONOO^−^) formation and related endothelial dysfunction [33]. It has been shown that a gradual reduction in the SOD level has been positively associated with different clinical grades of chronic periodontitis [34] and oral submucous fibrosis [35].

Among the three existing isoforms of SODs, the superoxide dismutase intracellular/cytoplasmic Cu/Zn SOD (SOD-1) is the primary isoform responsible for mitigating oxidative stress within the cytoplasm of cells [36].

Therefore, to better understand the mechanisms underlying the antioxidant capacity of the extracts and evaluate their potential effect in treating oral diseases, SOD-1 expression was investigated.

**Figure 5:**
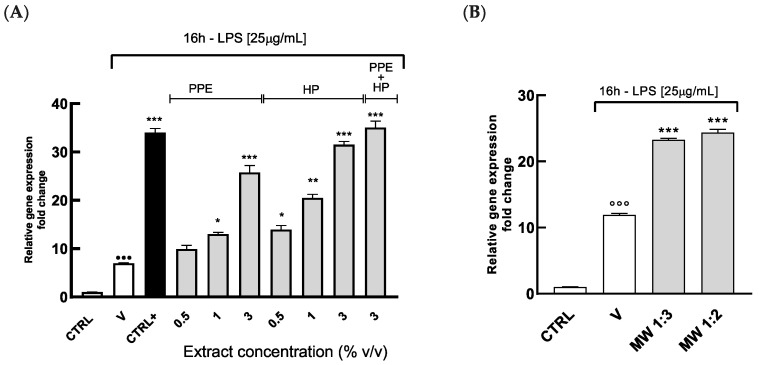
SOD-1 gene expression in HGECs treated with PPE and HP by using Real-Time PCR. HGECs were treated for 4 h with (**A**) PPE (range in content of punicalagin: 7.72–46.36 µg/mL), HP (range in content of lycopene: 0.12–0.75 µg/mL), PPE + HP (content of punicalagin: 46.36 µg/mL, content of lycopene: 0.75 µg/mL), and R (10µM) and (**B**) MW at the dilution 1:3 and 1:1 (range in content of punicalagin: 15.45–23.18 µg/mL, range in content of lycopene: 0.25–0.37 µg/mL), followed by treatment with LPS (25 µg/mL) for 16 h. SOD-1 expression was assayed by Real-Time PCR. Results are expressed as the mean ± SEM of at least three experiments. * *p* < 0.05, ** *p* < 0.01, and *** *p* < 0.001, significantly different from the LPS-treated HGECs; ^•••^/°°° *p* < 0.001, significantly different from control. Ctrl (control, untreated cells); V (cells treated with LPS); Ctrl+ (cells treated with R).

As shown in Figure 5A, SOD-1 expression increased in a dose-dependent manner after the treatment with the extracts (*p* < 0.05), except for PPE at 0.5% *v*/*v*, in HGECs challenged with LPS. Similarly, the mouthwash and the solution containing both extracts strongly increased SOD-1 expression in HGECs treated with LPS (*p* < 0.001) (Figure 5B). The effect of the combined HP and PPE treatment on SOD-1 gene expression in HGECs is comparable to that achieved with resveratrol, which is in line with previous studies showing its ability to modulate SOD activity in vitro and in vivo [37,38].

Data suggests that the extracts can partly counteract free radical damage thanks to the modulation of the intracellular antioxidant enzyme SOD-1. Moreover, the possible synergistic action of HP and PPE significantly enhances the overall antioxidant potential of these extracts and effectively increases SOD-1 levels.

Since the depletion in antioxidant systems contributes to the progression of oral pathologies [39], these data are of great interest. Indeed, the molecules within the extracts raised SOD-1 expression, thus partially restoring the defenses of the oral mucosa. This result is consistent with previous in vivo studies demonstrating that pretreatment with a solution enriched in ellagitannins or lycopene significantly increased depleted SOD levels in the gastric tissues of rats [40,41].

Additionally, the supplementary experiments showed that while the antioxidant activities of PPE and HP were slightly reduced in the presence of saliva, the effects of combined extracts remained significant. These results support their potential use for cosmetic applications under more physiologically relevant conditions (Supplementary Materials, Figure S1).

### 3.6. Anti-Inflammatory Effects of the Extracts on HGECs Injured with LPS

The inflammatory immune response is the key process in protecting periodontal tissues from bacteria and oral pathogens. However, a dysregulated inflammatory response is associated with the onset and the severity of periodontal and other oral diseases, leading to periodontal tissue destruction [42]. For example, high levels of tumor necrosis factor (TNF)-α can sustain the activation of secondary inflammatory mediators such as cyclooxygenase-2, and matrix metalloproteinases (MMPs), which contribute to the extracellular matrix degradation and bone resorption [43]. Moreover, a high level of Monocyte Chemoattractant Protein 1 (MCP-1) was found in crevicular fluids of adult patients with periodontitis, but not in those of healthy subjects. Studies suggest that MCP-1 may contribute to the marked infiltration of monocytes into tissues of periodontal patients, thus supporting the inflammatory state [44].

Therefore, to evaluate the anti-inflammatory effects of our extracts, we first examined the expression of TNF-α and MCP-1, in HGECs under LPS stimulation.

**Figure 6:**
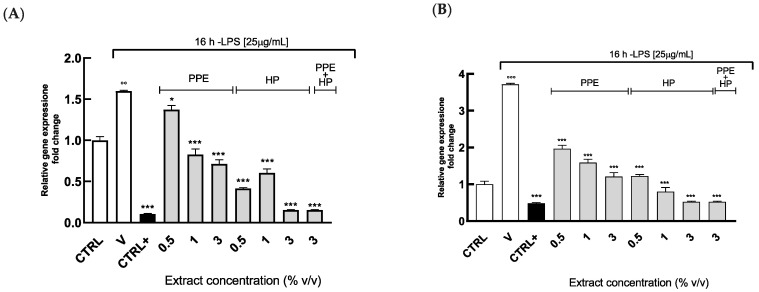
TNF-α and MCP-1 gene expressions in HGECs treated with PPE and HP by using Real-Time PCR. HGECs were treated for 4 h with PPE (range in content of punicalagin: 7.72–46.36 µg/mL), HP (range in content of lycopene: 0.12–0.75 µg/mL), PPE + HP (content of punicalagin: 46.36 µg/mL, content of lycopene: 0.75 µg/mL), and C (10 µM), followed by treatment with LPS (25 µg/mL) for 16 h. (**A**) TNF-α and (**B**) MCP-1 levels were assayed by Real-Time PCR. Results are expressed as the mean ± SEM of at least three experiments. * *p* < 0.05, *** *p* < 0.001, significantly different from the LPS-treated HGEPs; °° *p* < 0.01, °°° *p* < 0.001, significantly different from control. Ctrl (control, untreated cells); V (cells treated with LPS); Ctrl+ (cells treated with C).

Figure 6 shows that TNF-α and MCP-1 gene expression was significantly increased (*p* ˂ 0.001) in HGEPs stimulated by LPS compared to control, as expected. A 4 h pretreatment with the extracts (at all concentrations) significantly counteracted the LPS-induced TNF-α (*p* < 0.05) and MCP-1 (*p* ˂ 0.001) expression, suggesting their possible anti-inflammatory role. The expression of these inflammatory markers is reduced by the extracts in a dose-dependent manner, with a stronger effect given by HP. Additionally, at the concentration of 3% (*v*/*v*), HP alone or in combination with PPE showed effects comparable to those given by Curcumin treatment, a well-established anti-inflammatory agent known for its potent activity against these cytokines [23,45]. This suggests that HP, particularly when used synergistically with PPE, may serve as a reliable alternative ingredient in managing inflammatory responses.

The anti-inflammatory effect of PPE could be mainly associated with the ability of ellagitannins to modulate NF-κB signaling and decrease pro-inflammatory mediators, such as TNF-α, as already described in several cell line types [46,47]. Similarly, previous studies investigating the biological activities of tomato extracts highlighted the key role of lycopene in reducing inflammation through the modulation of the Nrf2 and NF-κB signaling pathways and downregulation of pro-inflammatory factors [48].

Overall, our findings were in line with the other reported studies [46,47,48], thus indicating the potential use of pomegranate and tomato by-product extracts in mitigating oral inflammation.

**Figure 7:**
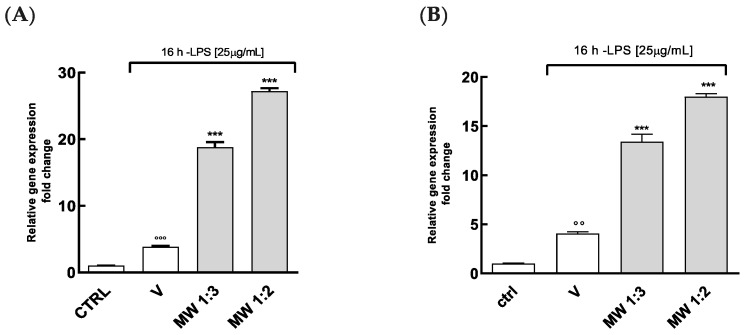
MCP-1 and TNF-α gene expressions in HGECs treated with MW formulations by using Real-Time PCR. HGECs were treated for 4 h with MW at the dilution 1:3 and 1:2 (range in content of punicalagin: 15.45–23.18 µg/mL, and range in content of lycopene: 0.25–0.37 µg/mL), followed by treatment with LPS (25 µg/mL) for 16 h. (**A**) TNF-α and (**B**) MCP-1 levels were assayed by Real-Time PCR. Results are expressed as the mean ± SEM of at least three experiments. *** *p* < 0.001, significantly different from the LPS-treated HGECs. °° *p* < 0.01, °°° *p* < 0.001, significantly different from control. Ctrl (control, untreated cells); V (cells treated with LPS).

In contrast, the mouthwash formulation showed the opposite effects, strongly increasing the TNF-α and MCP-1 gene expression (Figure 7).

Most likely, the co-presence of other components within the mouthwash formulation could lead to an antagonistic effect, reducing the anti-inflammatory potential of the active compounds or even supporting the acute inflammatory status.

For example, some preservatives may potentially increase the level of pro-inflammatory cytokines such as TNF-α, Interferon (IFN)-γ, Interleukin (IL)-1β, and Interleukin (IL)-6 [49]. Additionally, factors such as osmolarity, viscosity, and surface tension, often specifically designed to improve stability or to enhance user comfort, may influence the inflammatory response.

On the other hand, our results could be of interest since acute inflammation is still a protective response and an essential defense strategy, mostly concerning the oral cavity, which is exposed to several microbial agents.

Supplementary experiments showed that while the anti-inflammatory activities of PPE and HP treatment at low concentrations (0.5–1% *v*/*v*) were reduced when diluted in artificial saliva, their effects at higher concentrations (3%) and when combined were significantly improved. Additionally, saliva slightly reduced the pro-inflammatory effects of the mouthwash, minimizing potential contraindications. These findings support the extracts’ potential for cosmetic applications under physiologically relevant conditions (see Supplementary Materials, Figure S2).

These findings unveil the complexity of developing multi-component formulations, where possible synergistic or antagonistic interactions between ingredients can significantly influence biological outcomes. Further studies, such as reformulating with alternative, non-inflammatory excipients or examining their interactions, could help clarify the source of the pro-inflammatory effects and optimize the formulation for clinical use. It is important to balance these factors to minimize potential irritation while optimizing the desired therapeutic effects.

### 3.7. Antibacterial Effects of Extracts and Mouthwash Against S. mutans and S. sanguinis

The antibacterial properties of HP and PPE extracts were assayed in vitro against two oral bacteria, Streptococcus mutans and Streptococcus sanguinis, which are regarded as predominant species of the indigenous oral biota colonizing saliva and dental plaque, frequently associated with tooth surfaces free of caries.

**Figure 8:**
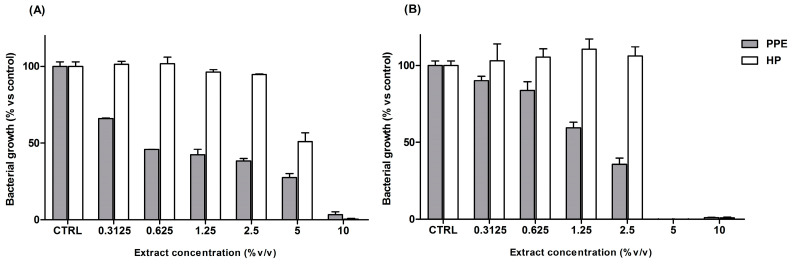
Antibacterial activity of the extracts (range: 0.3125–10%) assayed in *S. mutans* (**A**) and *S. sanguinis* (**B**), expressed as mean percentage values ± SEM relative to the control (CTRL, no treatment).

Figure 7 shows that bacterial proliferations were strongly affected by both extracts, with MIC values of 10% *v*/*v* for PPE and HP against S. mutans (Figure 8A) and 5% against S. sanguinis (Figure 8B), thus indicating an overall higher susceptibility of this latter strain. Although there were no differences in MIC values between the extracts, a different trend in the inhibitory potential was observed: while HP did not cause any change in bacterial growth at concentrations below the MIC, PPE exerted a strong dose-dependent effect on both bacteria.

The overall antibacterial potential of the mouthwash was evaluated against the same oral streptococcus species by using an agar cup diffusion test, instead of the microdilution assay, because the spectrophotometric measurements were strongly affected by the opalescence of this solution.

The effectiveness of the sample was demonstrated by the clear bacterial-free zone observed around the cup following a 24 h incubation at 37 °C. The diameters of the inhibitory zones were 24 ± 1 mm and 18 ± 1 mm, for S. mutans and S. sanguinis, thus suggesting the strong antibacterial properties of the mouthwash formulation.

## 4. Conclusions

This study highlights the potential use of PPE and HP extracts as active, sustainable ingredients obtained from agricultural waste for oral health applications. The extracts effectively reduced oxidative stress and inflammation in human gingival epithelial cells (HGECs), and PPE remarkably decreased bacterial growth. These results underscore an exciting opportunity within sustainable waste valorization, suggesting a viable path to transform food by-products into effective, natural ingredients for oral care products.

By upcycling agricultural waste, this research contributes to the broader effort of reducing agricultural chain waste while simultaneously addressing pressing environmental challenges. The integration of agrifood waste in the formulation of cosmeceuticals supports a circular economy model where food by-products are repurposed for new, value-added applications, driving sustainability in both oral health and waste management sectors.

While these preliminary findings are promising, several key scientific challenges and limitations remain critical to advancing this research. Firstly, although HGECs provide a valuable model for assessing cellular responses, they do not replicate the full complexity of the oral environment, including its diverse tissues and microbiome. In biological systems, PPE and HP’s bioavailability and chemical stability of PPE and HP could be altered by interactions with oral enzymes and microbial communities, potentially influencing their biological impact. Therefore, future studies should integrate more complex models, such as co-cultures, organoids, and ex vivo assessments, to allow a more comprehensive understanding of how these extracts perform under complex conditions.

Another critical consideration is the long-term stability of these bioactive compounds. Both polyphenols and lycopene are susceptible to degradation, which could limit the final product’s shelf life. Encapsulation and other preservation techniques may be necessary to ensure the sustained bioactivity and effectiveness of these extracts over time.

Looking forward, future research should focus on refining the formulation to improve tissue compatibility and stabilize bioactivity. This could include exploring the synergistic effects of combining polyphenols and carotenoids or blending these extracts with other natural antioxidants to enhance their overall impact. Advanced delivery systems, such as nanoencapsulation, also promise to improve bioavailability and ensure a controlled release of active compounds.

In summary, PPE and HP emerge as innovative candidates for antioxidant-rich oral health products, aligning with a circular economy model. The dual benefits of these extracts—promoting oral health while supporting sustainable practices—present an exciting frontier for research and development. By addressing the current challenges, PPE and HP could unlock a new generation of effective, eco-friendly oral care solutions that deliver both oral health and environmental benefits.

## Figures and Tables

**Table 1:** IC_50_ values were evaluated in HGEPs for the PPE, HP, PPE + HP, and MW using LPS as the pro-oxidant stimulus. Each value represents the mean ± SD of three replicate measurements. Resveratrol was used as a positive control (concentration range of 0.1–25 µM), exhibiting an IC_50_ value of 0.22 µM (corresponding to 0.05 ± 0.01 µg/mL).

4 h Treatment	IC_50_ (% *v*/*v*)	Punicalagin (µg/mL) ± SD	Lycopene (µg/mL) ± SD
PPE	0.36 ± 0.01	5.57 ± 0.04	-
HP	0.34 ± 0.02	-	0.08 ± 0.01
PPE + HP	0.23 ± 0.02	3.55 ± 0.02	0.05 ± 0.01
MW	0.25 ± 0.02	3.86 ± 0.03	0.06 ± 0.01

## Data Availability

The raw data supporting the conclusions of this article will be made available by the authors upon request.

## Supplementary Materials

The following supporting information can be downloaded at: https://www.mdpi.com/article/10.3390/antiox14010054/s1, Table S1: IC50 values were evaluated in HGEPs for the PPEsaliva, HPsaliva, and PPE+HPsaliva, using LPS as the pro-oxidant stimulus. Each value represents the mean ± SD of three replicate measurements. Figure S1: SOD-1 gene expression in HGECs treated with extracts prediluted in saliva, using Real Time-PCR assay. HGECs were treated for 4 h with (A) the extracts (HP, PPE, HP+PPE) and saliva-diluted extracts (HPsaliva, PPEsaliva, PPE+HPsaliva) in the range 0.5–3% *v*/*v*, and (B) MW and MWsaliva at the final concentration 1.5% *v*/*v* followed by treatment with LPS (25 μg/mL) for 16 h. Real Time-PCR assayed SOD-1 gene expression; results are expressed as mean ± SEM of at least three experiments. * *p* < 0.05, ** *p* < 0.01, *** *p* < 0.001 significantly different from the LPS-treated HGECs; °°° *p* < 0.001 significantly different from control. Ctrl (control, untreated cells); V (cells treated with LPS), Ctrl+ (cells treated with R, as the positive control). Figure S2. Using Real Time-PCR assay, TNF-α and MCP-1 gene expression was evaluated in HGECs treated with the extracts and the MW formulation, in the presence or absence of saliva. HGECs were treated for 4 h with saliva-untreated extracts (HP, PPE, HP+PPE) and saliva-treated extracts (HPsaliva, PPEsaliva, PPE+HPsaliva) in the range 0.5–3% *v*/*v*, as well as MW and MWsaliva at a final concentration of 1.5% *v*/*v*, and 10 μM Curcumin (C) followed by LPS (25 μg/mL) injuring for 16 h. (A,C) TNF-α and (B,D) MCP-1 expression levels were assayed by Real Time-PCR. Results are showed as mean ± SEM of at least three experiments. * *p* < 0.05, *** *p* < 0.001 significantly different from the LPS-treated HGEPs; °° *p* < 0.01, °°° *p* < 0.001, significantly different from control. Ctrl (control, untreated cells); V (cells treated with LPS), Ctrl+ (cells treated with C, as the positive control). References [21,50,51,52] are cited in the supplementary materials.
