# Supplementary material for: Anti-Inflammatory, Antioxidant and Antibacterial Properties of Tomato Skin and Pomegranate Peel Extracts: A Sustainable Approach for Oral Health Care
Source: Antioxidants (Basel). 2025 Jan 5;14(1):54. doi: 10.3390/antiox14010054 (PMC11762152; doi:10.3390/antiox14010054)
Supplement: Supplementary file 1 [file antioxidants-14-00054-s001.zip › antioxidants-3367930-supplementary.pdf]

Supplementary Materials

Methods

Preparation of PPE and HP extracts diluted in Artificial Saliva

Artificial saliva for Pharmaceutical Research SAE0149 (Sigma, St. Louis, MO, USA) was used to mimic natural saliva's chemical composition and physical properties. Pomegranate peel extract (PPE) and Hydropom (HP) were prepared at concentrations ranging from 1.5% to 9% (*v/v*) in the working artificial saliva solution (pH 6.8). A combination of HP and PPE at a final concentration of 9% was also prepared in the same condition. Then, the saliva solutions containing PPE, HP, and HP+PPE were diluted in the cell culture medium at a 1:3 ratio (saliva: medium) and pre-incubated for 30 min at 37 °C to improve interaction with the cellular environment. According to the dilutions performed, the final concentrations of extracts in the working solutions were 0.5-3% (*v/v*), and the extracts were marked as follows: PPE_{saliva}, HP_{saliva}, and $HP+PPE_{saliva}$.

MW formulation, containing both HP and PPE at the concentration of 6% (*v/v*), was diluted with saliva at a 1:2 ratio (saliva: medium). Before the experiment, the saliva solution containing MW was diluted in the cell culture medium in the range of 1:2-1:4 and pre-incubated for 30 min at 37 °C. The final concentrations of extracts in the working solution ranged from 0.75 to 1.5% (*v/v*) and were marked as MW_{saliva}.

Antioxidant Activity Assay

The antioxidant activity of the extracts was measured using a chemiluminescence assay previously described by Calabria et al. [1] to evaluate the intracellular H_2O_2 production in Human gingival epithelial cells (HGECs). HGECs were seeded in transparent 96-well plates at 10 x 10^4 cells/well density and incubated at 37 °C, 5% CO_2. After 24 h, cells were treated for 4 h with different dilutions of PPE_{saliva}, HP_{saliva} and $HP+PPE_{saliva}$ (concentration range 0.5%–3% *v/v*) and MW_{saliva} (concentration range 0.75 - 1.5% *v/v*). Untreated cells served as baseline controls.
After the treatments, the medium was replaced with 50 µL of fresh medium, and 50 µL of the CL probe solution (final concentration of 5 µM/well) was added to each well and incubated for 20 min at 37 °C. After the incubation, 50 µL of LPS at a fixed concentration (final concentration of 25 µg/mL) were added to each well. CL emission signal was monitored using a Luminoskan™ Ascent luminometric plate reader. The whole assay was performed at 37 °C. The dose-response curve was obtained by plotting the CL signal versus the concentration of the extracts or mouthwash treatments.

RNA Extraction HGECs were seeded in six-well plates (1 × 10^6 cells/well) and grown at 37 °C, 5% CO_2. The following day, cells were pretreated for 4 h with serial dilutions of PPE, HP, PPE_{saliva}, HP_{saliva} (at the final concentration of 0.5-3% *v/v*), PPE+HP and $PPE+HP_{saliva}$ (at the final concentration of 3% *v/v*) and MW and MW_{saliva} (final concentration range 1.5%). Treatments with resveratrol (R) (10µM) and curcumin (C) (10 µM) were used as positive controls as reference antioxidant and anti-inflammatory compounds, respectively. After treatment, cells were stimulated with LPS (25 µg/mL) for 16 h. Total RNA was extracted using the RNeasy Mini Kit according to the manufacturer's instructions. RNA quantity and quality were determined using a Nanodrop 1000 spectrophotometer (Thermo Fisher Scientific).

Real-Time PCR
Total RNA (25 ng) was reverse transcribed and analyzed using the Power SYBR™ Green RNA-to-CT™ 1-Step Kit. Reaction conditions were conducted on a QuantStudio1 Real-Time PCR System (Thermo Fisher Scientific, Waltham, MA, USA), with an initial reverse transcription of 10 min at 45 °C, then 2 min at 95 °C, followed by 40 cycles of amplification (95 °C for 5 s and 60 °C for 20 s) and evaluated by QuantStudio 1 Real-Time PCR System Software. Primer concentration was 50 µM. The following human primers were used:

RPL13A forward 5′- CACCCTGGAGGAGAAGAGGA -3′, reverse 5′- CCGTAGCCTCATGAGCTGTT -3′.

SOD-1 forward 5′-AGGCATGTTGGAGACTTGGG -3′, reverse 5′- TGCTTTTTCATGGACCACCAG -3′;

TNF-α forward 5′- CCATGTTGTAGCAAACCC -3′, reverse 5′- GAGTAGATGAGGTACAGGC -3′.

MCP-1 forward 5′- GATCTCAGTGCAGAGGCTCG -3′, reverse 5′- GGTCTTGAAGATCACAGCTTCTT -3′.

Changes in gene expression levels were analyzed by the $2^{-\Delta\Delta Ct}$ formula and the RPL13A (ribosomal protein L13-A) was used as the reference gene.

Statistical Analysis

Results are expressed as mean ± SD of at least three independent experiments. The existence of differences between any pairs of means was determined by one-way ANOVA, followed by Tukey's HSD post-hoc test to assess the pairwise significance of differences between groups. GraphPad Prism v. 6.0 (GraphPad Software, Inc., La Jolla, CA, USA) was used to plot the experimental data and for the least-squares fitting of calibration, dose-response graphs (for evaluation of IC_{50} values), and assay comparison graphs. A P value < 0.05 was considered statistically significant.

Results

Antioxidant Efficacy of PPE and HP Extracts diluted in saliva, in Human Gingival Epithelial Cells

In this study, we aimed to evaluate the efficacy of the extracts previously diluted in artificial saliva. The antioxidant capacity was measured using a chemiluminescence bioassay to selectively detect the intracellular production of H_2O_2. We specifically compared the antioxidant effects of the extracts in their original form to those diluted in artificial saliva at equivalent concentrations. As shown in Table S1, the IC50 values obtained from the extracts and mouthwash diluted in saliva were higher than those of untreated samples (data reported in the main test in paragraph 3.4).

Table S1: IC_{50} values were evaluated in HGEPs for the PPE_{saliva}, HP_{saliva}, and $PPE+HP_{saliva}$, using LPS as the pro-oxidant stimulus. Each value represents the mean ± SD of three replicate measurements.

**4 h treatment**	**IC_{50} (% *v/v*)**
PPE_{saliva}	0.58 ± 0.03
HP_{saliva}	0.53 ± 0.04
$PPE+HP_{saliva}$	0.40 ± 0.04
MW_{saliva}	0.46 ± 0.07

The reduction of H_2O_2 scavenging ability of the extracts and the mouthwash previously diluted in saliva suggests a diminished antioxidant efficacy, which can be attributed to several factors. Firstly, the saliva ionic composition and salinity could negatively affect the stability of key bioactive compounds, such as polyphenols and lycopene. Moreover, the ions may lead to interactions that alter the solubility of these compounds, thereby reducing their effectiveness in neutralizing ROS [2,3]. Such interactions could potentially reduce their capacity to scavenge free radicals effectively. Despite a reduction in the extracts and mouthwash effectiveness, in the presence of saliva, they still exhibit significant antioxidant properties.

Next, to better evaluate their antioxidant ability, we examined the expression levels of SOD-1 in HGECs, after 4 h treatments with the extracts (HP, PPE, HP+PPE) and saliva-diluted extracts (HP_{saliva}, PPE_{saliva}, $PPE+HP_{saliva}$) in the range 0.5-3% (*v/v*), and MW and MW_{saliva} using the final concentration of 1.5% (*v/v*). Resveratrol (R) at 10 μM was included as the positive control.

As shown in Figure S.1A, treatments with PPE, HP, and HP+PPE exhibited a dose-dependent upregulation of SOD-1 gene expression, with HP demonstrating the most promising effect. Similarly, MW treatment significantly increases the SOD-1 gene expression (Figure S.1B). The upregulation of SOD-1 is significant as it plays a crucial role in the cellular defense against oxidative stress by catalyzing the dismutation of superoxide radicals into hydrogen peroxide and molecular oxygen.

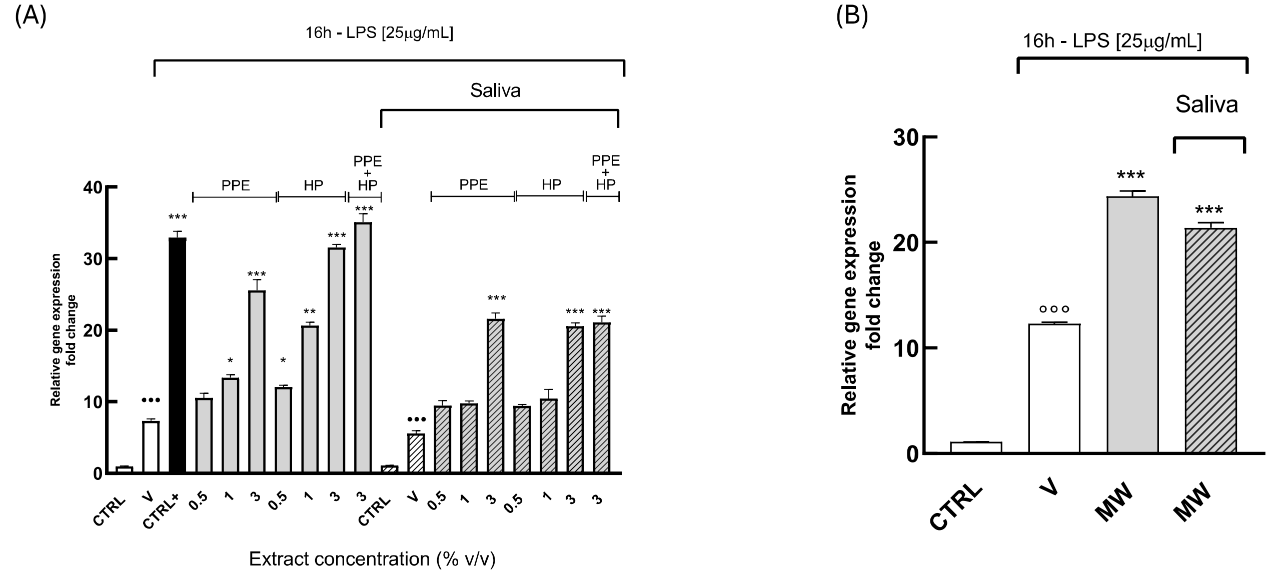

Figure S1. SOD-1 gene expression in HGECs treated with extracts prediluted in saliva, by using Real Time-PCR assay. HGECs were treated for 4 h with (A) the extracts (HP, PPE, HP+PPE) and saliva-diluted extracts (HP_{saliva}, PPE_{saliva}, PPE+HP_{saliva}) in the range 0.5-3% v/*v*, and (B) MW and MW_{saliva} at the final concentration 1.5% *v/v* followed by treatment with LPS (25 μg/mL) for 16 h. SOD-1 gene expression was assayed by Real Time-PCR. Results are expressed as mean ± SEM of at least three experiments. * $p < 0.05$, ** $p < 0.01$, *** $p < 0.001$ significantly different from the LPS-treated HGECs; °°° $p < 0.001$ significantly different from control. Ctrl (control, untreated cells); V (cells treated with LPS), Ctrl+ (cells treated with R, as the positive control).

Regarding treatments with extracts pre-diluted in saliva, a significant upregulation of SOD-1 gene expression was observed just for HP_{saliva}, and PPE_{saliva} at the highest concentration (3% *v/v*), PPE+HP_{saliva}, and MW_{saliva}. In contrast, the extracts in the concentration range between 0.5-1% (*v/v*) did not significantly enhance SOD-1 levels. These findings suggest that although saliva may affect the action and stability of the active compounds present in the extracts, increasing their concentration can counteract this effect and maintain their ability to boost antioxidant defenses. Indeed, the antioxidant activity of MW_{saliva} treatment was preserved under these conditions suggesting that the extracts remain valuable ingredients for counteracting oxidative stress.

Anti-Inflammatory Efficacy of PPE and HP Extracts diluted in saliva, in Human Gingival Epithelial Cells

To evaluate the anti-inflammatory potential of the extracts, we analyzed the expression of TNF-*α* and MCP-1 genes in HGECs. HGECs were treated for 4 h with the extracts (HP, PPE, HP+PPE) and saliva-diluted extracts (HP_{saliva}, PPE_{saliva}, PPE+HP_{saliva}) in the range 0.5–3% *(v/v)*, as well as MW and MW_{saliva} at a final concentration of 1.5% (*v/v*). Curcumin (C) at 10 μM was included as the positive control. Following treatments, the cells were exposed to LPS (25 μg/mL) for 16 hours to induce the inflammatory process.

As shown in Figures S2A and S2B, PPE, HP, and HP+PPE treatments significantly reduced LPS-induced TNF-*α* and MCP-1 gene expressions ($p < 0.5$), highlighting their potential as anti-inflammatory agents. Among these, HP treatment demonstrated a stronger modulation of inflammatory markers compared to PPE treatment across all concentrations. Saliva-diluted extracts (PPE_{saliva} and HP_{saliva}) effectively counteracted TNF-*α* and MCP-1 gene expressions only at the highest concentration (3%) and when combined (PPE+HP_{saliva}). Lower concentrations (0.5% and 1% v/v) did not exhibit significant effects.

These data suggest that saliva may interfere with the stability and activity of the active compounds and, thus affecting the anti-inflammatory capacity of the extracts, particularly at lower concentrations.

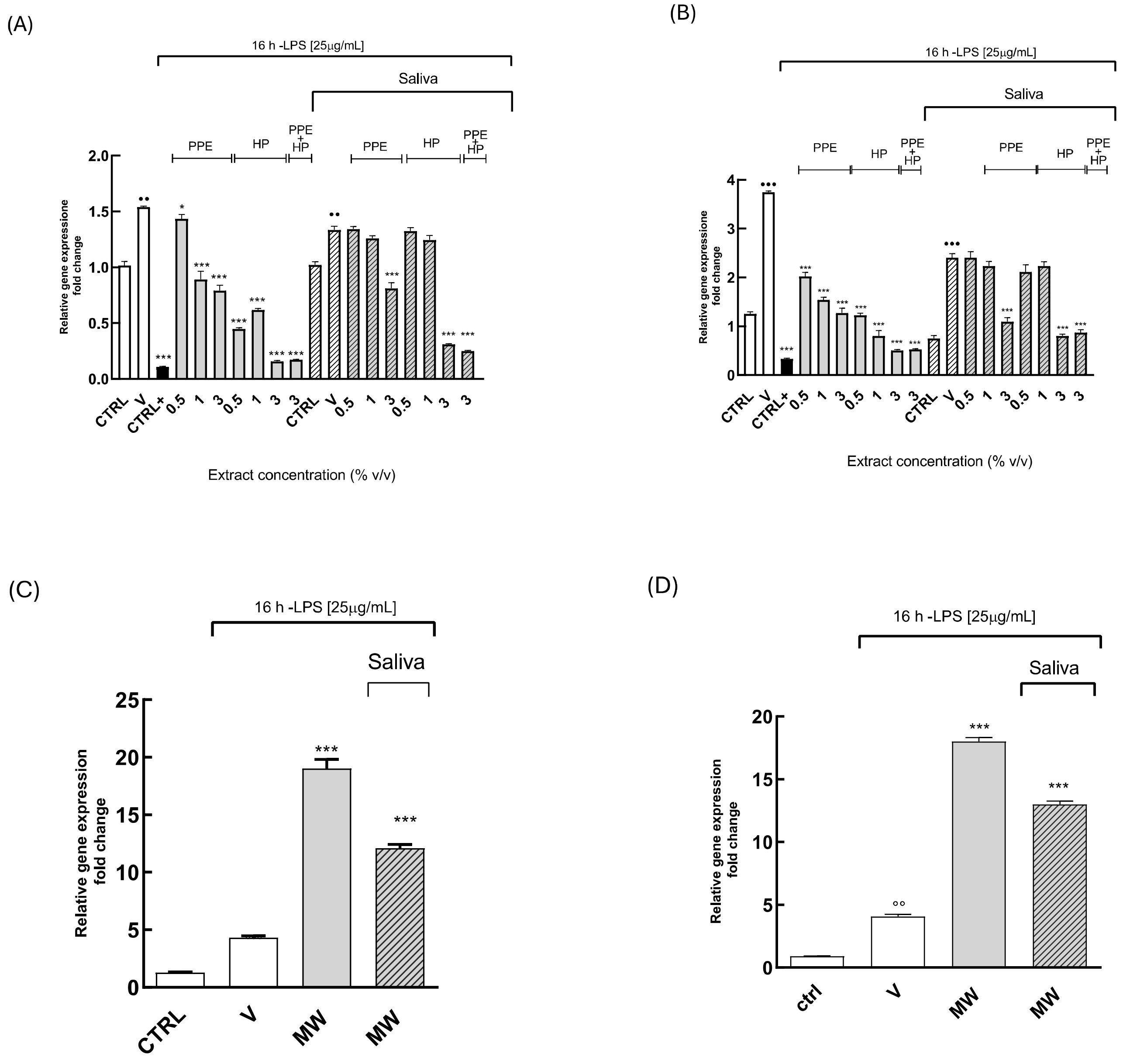

FIGURE S2. TNF-*α* and MCP-1 gene expression in HGECs treated with the extracts and the MW formulation, in the presence or absence of saliva, by using Real Time-PCR assay. HGECs were treated for 4 h with saliva-untreated extracts (HP, PPE, HP+PPE) and saliva-treated extracts (HP_{saliva}, PPE_{saliva}, PPE+HP_{saliva}) in the range 0.5–3% *v/v*, as well as MW and MW_{saliva} at a final concentration of 1.5% v/v, and 10 μM Curcumin (C) followed by LPS (25 μg/mL) injuring for 16 h. (A, C) TNF-*α* and (B, D) MCP-1 levels were assayed by Real Time-PCR. Results are expressed as mean ± SEM of at least three experiments. * $p < 0.05$, *** $p < 0.001$ significantly different from the LPS-treated HGEPs; °° $p < 0.01$, °°° $p < 0.001$, significantly different from control. Ctrl (control, untreated cells); V (cells treated with LPS), Ctrl+ (cells treated with C, as the positive control).

These findings emphasize the importance of the concentration of the treatment in achieving optimal anti-inflammatory effects. Nevertheless, the significant modulation of inflammatory markers by PPE and HP at higher concentrations or when combined underscores that they still remain powerfull anti-inflammatory agents.

In contrast, MW and MW_{saliva} treatments significanly increased TNF and MCP-1 gene expressions, which can be attributed to factors such as the presence of preservatives (as detailed in the main text, paragraph 3.7). However, treatment with MW_{saliva} slightly reduces these pro-inflammatory effects. This reduction may be attributed to the ionic composition of artificial saliva, including sodium and potassium concentrations that may provide a buffering effect [4], reducing irritation caused by mouthwash components and contributing to a less inflammatory environment in the oral cavity.

Conclusion

Despite possible interactions with components in artificial saliva, the extracts maintained their beneficial biological activities at the concentrations used in the final mouthwash formulation. These results support the use of these extracts in oral care products, demonstrating their potential effectiveness in promoting oral health.

References

1. Calabria, D.; Guardigli, M.; Mirasoli, M.; Punzo, A.; Porru, E.; Zangheri, M.; Simoni, P.; Pagnotta, E.; Ugolini, L.; Lazzeri, L.; et al. Selective Chemiluminescent TURN-ON Quantitative Bioassay and Imaging of Intracellular Hydrogen Peroxide in Human Living Cells. *Anal Biochem* 2020, *600*, 113760, doi:10.1016/j.ab.2020.113760.
2. Jabbari, M.; Gharib, F. Solvent Dependence on Antioxidant Activity of Some Water-Insoluble Flavonoids and Their Cerium(IV) Complexes. *J Mol Liq* 2012, *168*, 36–41, doi:10.1016/j.molliq.2012.02.001.
3. Cristea, E.; Sturza, R.; Jauregi, P.; Niculaua, M.; Ghendov-Moșanu, A.; Patras, A. Influence of PH and Ionic Strength on the Color Parameters and Antioxidant Properties of an Ethanolic Red Grape Marc Extract. *J Food Biochem* 2019, *43*, e12788, doi:10.1111/jfbc.12788.
4. Humphrey, S.P.; Williamson, R.T. A Review of Saliva: Normal Composition, Flow, and Function. *J Prosthet Dent* 2001, *85*, 162–169, doi:10.1067/mpr.2001.113778.
